# GLOBAL ASSESSMENTS OF END-OF-LIFE CARE: HOW ARE THESE CARE MEASURES PATTERNED BY PROXY RELATIONSHIP TO DECEDENT?

**DOI:** 10.1093/geroni/igae098.2029

**Published:** 2024-12-31

**Authors:** Kafayat Mahmoud, Deborah Carr, David Ekerdt

**Affiliations:** Boston University, Boston, Massachusetts, United States; Boston University, Boston, Massachusetts, United States; University of Kansas, Lawrence, Kansas, United States

## Abstract

A “good death” is one in which the patient’s physical, emotional, and spiritual needs are met, they are treated with dignity and respect by health care providers, and treatment preferences are honored. Despite policies to help improve death quality, including support for doctor-patient end-of-life consultations as part of the Affordable Care Act, it is unclear whether death quality has improved, nor whether particular people are vulnerable to poor quality deaths. Researchers rely heavily on proxy-reported measures of end-of-life, however it is unclear whether these reports differ by one’s relationship to the decedent. We use 13 waves of the National Health and Aging Trends Study (NHATS), and polychoric factor analysis and multinomial and logistic regressions to identify the contributions of 10 specific dimensions of end-of-life care to overall quality of care evaluations, and how these assessments are affected by the proxy’s relationship to the decedent, after controlling for socio-demographic and health characteristics of decedents. Overall care ratings are highly correlated with perceptions of personal care needs met, respectful treatment, being informed about care, and care coordination. Physical and emotional symptoms, care wantedness, and decisions made without patient input have weaker associations with the overall care assessment. Paid caregiver proxies give more positive assessments of care relative to spouses and children of decedents. Adult children proxies offer more positive assessments of care decisions and wantedness, whereas spouse proxies report better management of physical and emotional symptoms. We discuss the implications for data collection as well as health care practice.

